# Progress in State Medicine

**Published:** 1901-11-02

**Authors:** 


### PROGRESS IN STATE MEDICINE.

### SEWAGE AND ITS TREATMENT.

EXPERIENCE still seems to point to the fact that the purification of sewage through the agency of bacteria is the most rational, economic and effective method.[1] Scott-Moncrieff, experimenting in 1891, erected at Ashtead an apparatus for dealing with the sewage of a household containing from 10 to 12 persons. At first it consisted only of a "cultivation tank," 2½ feet wide, 10 feet long, and about 3 feet deep at its deepest part. The entire sewage and waste water finds its way into one end of the tank. The liquid portion rises through a perforated grating at the bottom of the tank, and then through a layer of large flints until it reaches the level of the outflow pipe, which is about 2 inches above the level of the invert of the drain. The mean depth of the filtering material is about 14 inches, and the cubic space of the channel beneath the grating only about 5 cubic feet. It was found that, allowing 8 to 4 cubic feet of flints to each inhabitant, there was almost complete liquefaction of the solid matter and that the sludge was a negligible quantity. The compound nitrogenous substances are broken down to a great extent into nitrogen and free ammonia, and the carbon compounds into carbonic acid and water. Some years were spent in attempts to produce the maximum oxidation of the effluent. At last a very simple apparatus was devised by which the "nitrification" is carried out to an extent which has not been attained by any other method. Nine wooden boxes, with perforated bottoms, each of them 2 feet long by 7 inches wide and 7 inches deep, filled with coke about the size of beans, are arranged one over the other, with a clear space of 2 inches between each. The effluent from the cultivation tank is evenly distributed over the surfaces of the coke in the topmost boxes by V-shaped troughs, so arranged that they discharge their contents automatically at intervals of about every seven minutes. This system is automatic, requires no skilled supervision or repair. Rideal states with regard to the final effluent from the nitrifying trays that it could be easily "finished" by a fine filter without fouling the latter, or could be beneficially applied to a small area of land, or mixed with a river of small volume, not only without pollution, but possibly with an actual benefit to the stream. An installation of this system is now dealing with 16,000 gallons daily of extraordinarily strong sewage from Caterham Barracks. The final effluent escapes as a clear colourless fluid, free from smell and non-putrescible, even when incubated for some days. It has a high fertilising power when applied to land. It is claimed that both anaërobic and aërobic conditions are provided in this system, the former in the "cultivation tank," the latter in the "nitrifying trays." Further, the process appears to be capable of conserving nearly the whole of the original nitrogen in the sewage, instead of dissipating it in gaseous forms, thus greatly increasing its fertilising properties.

In the Candy[2] system of sewage purification the sewage is first passed through a small detritus tank which retains the grosser particles, and then through patent "Candy-Caink Sprinklers" on to two "anaërobic" beds in succession, by a process of continuous filtration. The sprinklers are two hollow perforated arms, attached to a central basin, which runs on special bearings. The sewage is led into the central basin, flows into the arms, and issuing therefrom in jets, due to the eight inches or so of head, causes the whole sprinkler to revolve; thus the sewage is showered evenly upon every part of the bed. The size of the perforations being progressively arranged according to their distance from the centre, every square inch of the bed receives its proper dose and no more. By means of a syphon, discharging at regular intervals, the sprinklers are made to act intermittently, thus allowing a continual current of fresh air to pass through the bed. There are two filter beds, circular in shape, containing coarse particles of broken brick, etc. At first the upper bed was worked as an anaërobic tank, but it was subsequently converted into an aërobic filter, differing from the other in containing coarser particles. Most excellent results are recorded with this system at Reigate. Although the beds have been working without interruption since installation no signs of choking, either of the beds or sprinklers, have been detected; no labour or alterations have been required.

Houston,[3] writing on the "Bacterial Treatment of London Sewage," quotes the conclusions arrived at by Clowes, viz., that bacterial treatment is superior to the present chemical precipitation in that it requires no chemicals, produces no offensive sludge, and removes the whole of the suspended matter instead of only about 80 per cent. Further, the resultant liquid is entirely free from objectionable smell, and does not become foul when it is kept; it further maintains the life of fish. The difficulty at first experienced by the choking of the beds through the heavy nature of the London sewage has been overcome by a simple and rapid sedimentation of the crude sewage. Houston points out that a more extensive bacteriological knowledge of crude sewage is required as a preparatory step to the study of effluents from bacterial beds. From a number of experiments he states that in crude sewage the number of bacteria causing liquefaction of gelatin is likely to be not less than 1,000,000 per c.c., and the number of spores of aërobic bacteria between 300 and 400 in 1 c.c. Although the effluents from the baterial beds showed a diminution in the numbers of liquefying spores, as compared with crude sewage, the reduction was immaterial considering the large numbers still remaining. Barking and Crossness crude sewage injected into guinea-pigs usually caused their death in 24 to 70 hours. Sometimes the effluents from the coke beds are more pathogenic than the raw sewage, but usually a larger dose of the effluents is required to produce a fatal effect. After heating the crude sewage, or effluent, to 100°C. for one hour, large doses may be injected without producing a fatal effect. If either be filtered through a sterilised Pasteur's filter, very large doses of the filtrate fail to produce a pathogenic effect. The filtrate on the coke beds, when examined bacteriologically, was found to contain 1,800,000 bacteria per grm. As compared with crude sewage this deposit contained more spores of B. Enteritidis Sporogenes, and fewer B. Coli. Further experiments showed that the cholera bacillus and staphylococcus pyogenes aureus are capable of retaining their vitality in Crossness sewage, in competition with the very numerous bacteria normally present in the liquid, for a considerable time. This seems to point to the fact that the saprophytic bacteria normally present in sewage would not suffice for the destruction of pathogenic species, either in the sewers, or later in the bacterial beds. He is strongly of opinion that if the effluent contains streptococci in practically unaltered numbers or even in reduced numbers (where such reduction is only in the same proportion as in the other microbes) then such effluent is as regards danger to health, no better in the first case than raw sewage, and no safer in the latter than crude sewage slightly diluted, but otherwise unaltered in its possible capacity of giving rise to disease. The modern systems of sewage disposal cannot be depended upon to remove the B typhosus and allied organisms. When an effluent is turned into a stream to be used for drinking purposes, the bacteriological results are of more importance than the chemical; but when the river is not a potable one, the chemical state of the effluent is of primary, and the biological of secondary, importance. His final conclusions from the Barking and Crossness experiments are that the effluents from the bacterial beds cannot reasonably be assumed to be more safe in their possible relation to disease than raw sewage. Attention [4] is called to the fact that although the germ theory of infectious diseases is universally accepted, little attention has been devoted to the possibility of destroying the pathogenic bacteria in sewage and sewage effluents; efforts being principally directed to the arrest of putrefaction. These do not necessarily involve the destruction of bacteria. It is conceivable that the "septic tanks" and "bacterial beds" afford not only ample nutrition, but various other conditions most congenial to the multiplication and vigorous vitality of pathogenic germs. The dangerous potentiality of sewage depends not on its chemical ingredients, not on the proportion of the putrescent materials, nor on the capacity for undergoing putrefaction, but on the pathogenic bacteria which it contains. Efforts must be made to ascertain whether, for example, typhoid bacilli survive long in sewage, and whether they are diminished or destroyed by the various processes used for sewage purification. Andrews and Laws report that the chance of detecting the B. typhosus in sewage was "so remote as to be almost infinitesimal." This bacillus, when introduced into sterilised sewage, dies after a few days or one or two weeks. This length of time is obviously sufficient to allow the bacilli to be carried to remote distances and become sources of fresh infection. Horrocks detected them living in sterilised sewage 60 days after introduction. Very great reduction in the number of bacilli in effluents is recorded in those cases where secondary beds are in use. Communities should substitute the bacterial system for that of precipitation and sedimentation, and in view of the fact that still further means of purification are required should early acquire land in the immediate neighbourhood, as insisted upon by the Local Government Board, for this purpose. Water for domestic purposes drawn from a source likely to become infected should be filtered in large sand filters which, if properly attended to, would remove the bacteria to such an extent that the number in the filtrate would not exceed more than 1 or 2 per cent. of the total number present before filtration. Most stringent precautions must be taken to prevent bacilli gaining access to the sewers direct from the bedsides of patients. Nurses hold the first line of defence. The effluents from the bacterial beds might be sterilised, the chief objection being one of expense. Where the volume of sewage is large in proportion to the volume of a potable stream into which it discharges, the effluent ought undoubtedly to be sterilised until it can be shown that it is purified, not only of putrescible matter, but also of all pathogenic bacteria present. M. Felix Launay[5] points out that more has been done in England than in France to prevent pollution of rivers from sewage. Considerable evil had resulted from the belief that rivers were capable of purifying the sewage they received. He mentions that Pettenkofer had shown that the sewage of Munich was purified by the River Isar, but a river must be in proportion to the discharge of sewage which was rarely the case. Owing to the differing nature of rivers and sewage no hard and fast rule could be laid down; the safest rule being to prohibit any crude sewage being discharged into any river. The utilisation of sewage for agricultural purposes is recognised as the best process of purification. Failing suitable soil artificial means must be employed, and the Dibdin process, or the Cameron process seemed to give the best promise of success. In one way or another it is now possible to prevent pollution of rivers.

[1] Brit. Med. Journ.. Sept. 29, 1900. [2] Ibid., Oct. 13, 1900. [3] Edin. Med. Journ., Feb., 1901. [4] Brit. Med. Journ., April 13, 1901. [5] Lancet, Aug. 25, 1900.

